# Selective non-operative management of traumatic liver injury in Palestine: a retrospective single-center cohort study

**DOI:** 10.3389/fmed.2026.1878227

**Published:** 2026-09-14

**Authors:** Abdallah Alem, Ali Shakhshir, Shaymaa Kabiya, Tawfeeq Al-Shobaki, Amjad Awwad

**Affiliations:** 1 Department of Clinical Medicine, Faculty of Medicine, Arab American University, Jenin, Palestine; 2 Scientific Research Committee, Faculty of Medicine, Arab American University, Jenin, Palestine; 3 Faculty of Medicine, Arab American University, Jenin, Palestine; 4 Department of General Surgery, Ibn Sina Specialized Hospital, Jenin, Palestine

**Keywords:** gunshot injury, hepatic trauma, liver injury grade, non-operative management, Palestine, retrospective cohort, traumatic liver injury

## Abstract

**Background:**

Non-operative management (NOM) is the preferred strategy for appropriately selected patients with traumatic liver injury, but evidence from Palestine and other under-represented trauma settings remains limited. This study described initial management selection, in-hospital outcomes, and resource use among patients with traumatic liver injury at a Palestinian trauma center.

**Materials and methods:**

This retrospective observational cohort study included consecutive patients with confirmed traumatic liver injury managed at Ibn Sina Specialized Hospital, Jenin, Palestine, between January 2021 and December 2025. Patients were classified according to initial management as NOM or initial laparotomy. The primary outcome among patients initially managed non-operatively was NOM success, defined as survival without delayed operative conversion during the index hospitalization. A secondary composite endpoint, termed an unfavorable NOM course, included biliary leak, any in-hospital complication, any documented endoscopic or drainage procedure, delayed surgery, or in-hospital death. Individual components were also reported separately.

**Results:**

A total of 58 patients were included; 48 (82.8%) underwent initial NOM and 10 (17.2%) underwent initial laparotomy. All 27 penetrating injuries were gunshot wounds. NOM success was observed in 48 of 48 patients (100%; exact 95% CI, 92.6%-100.0%), with no delayed operative conversion or in-hospital death after initial NOM. An unfavorable NOM course occurred in 6 of 48 patients (12.5%) and was driven by documented endoscopic or drainage procedures in four patients and in-hospital complications in three; component counts overlapped. Higher-grade AAST III-IV injury was associated with an unfavorable NOM course compared with AAST I-II injury (41.7% vs. 2.8%, *P* = 0.002), as well as greater complication burden, procedure use, ICU stay, hospital stay, and transfusion requirements. All unfavorable NOM courses occurred after gunshot injury.

**Conclusion:**

No NOM failure or in-hospital death was observed among the selected patients initially managed non-operatively. However, the small cohort, limited event count, index-hospitalization follow-up, and absence of consistently recorded physiologic selection variables preclude definitive conclusions regarding safety or comparative effectiveness. Higher-grade AAST III-IV and gunshot injuries were associated with more complicated in-hospital NOM courses and greater resource use.

## Introduction

Traumatic liver injury is a frequent and potentially life-threatening abdominal injury because of the liver's size, location, and vascularity (1, 2). Hepatic trauma may be accompanied by major hemorrhage, transfusion requirements, bile leak, intra-abdominal collections, and sepsis (3). Outcomes are also influenced by associated intra-abdominal and extra-abdominal injuries rather than liver injury alone (4, 5).

Historically, suspected solid-organ injury commonly prompted exploratory laparotomy. Improvements in computed tomography (CT), critical care, interventional techniques, and therapeutic endoscopy have supported a shift toward selective non-operative management (NOM), particularly for patients without an immediate operative indication (1, 2, 6, 7). Contemporary guidance emphasizes that NOM depends on appropriate selection, serial clinical and laboratory assessment, access to imaging and blood products, and the ability to escalate promptly to surgery or adjunctive procedures when required (1, 8). Reported NOM success rates are generally high in selected cohorts, although estimates vary with injury grade, mechanism, case mix, and the availability of adjunctive care (3, 9).

Despite high overall success rates, some patients experience NOM failure or a clinically complicated course. Reported correlates include higher American Association for the Surgery of Trauma (AAST) grade, hemoperitoneum or active bleeding, transfusion requirement, and the need for angioembolization (10, 11). Higher-grade injuries and complex penetrating trauma may be accompanied by greater risks of hemorrhagic, biliary, and infectious complications (12, 13). Associated gastrointestinal, pancreatic, mesenteric, and extra-abdominal injuries may further influence management and outcomes (14).

Most contemporary evidence is derived from large trauma systems and well-resourced referral centers (15, 16). Evidence from conflict-affected and under-resourced regions remains comparatively limited, and local injury patterns, referral pathways, and access to advanced adjunctive care may differ (17). Palestine remains under-represented in the liver-trauma literature. This study therefore aimed to describe the use, feasibility, and short-term clinical outcomes of initial NOM for traumatic liver injury at a Palestinian trauma center, with particular attention to NOM success, the components of an unfavorable in-hospital NOM course, injury grade, and mechanism of injury.

## Methodology

### Study design and setting

This retrospective observational cohort study evaluated patients with traumatic liver injury managed at Ibn Sina Specialized Hospital, Jenin, Palestine, between January 2021 and December 2025. The study was reported in accordance with the Strengthening the Reporting of Observational Studies in Epidemiology (STROBE) statement for cohort studies. Cases were identified from Surgery Department admission logs, operative records, radiology records, and medical charts. Data were reviewed retrospectively using a structured data-abstraction form based on predefined variables and outcome definitions.

### Participants and eligibility criteria

All consecutive patients with confirmed traumatic liver injury during the study period were considered eligible, regardless of age or sex. Both blunt and penetrating mechanisms were included. Liver injury was confirmed by radiologic findings and/or intraoperative documentation. Patients were included when the record allowed confirmation of liver injury and classification of the initial management strategy; records were excluded when either could not be established. All penetrating injuries in the analytic cohort were documented as firearm-related gunshot wounds; no stab injuries were represented. The retained analytic dataset contained the 58 eligible included cases, but counts of records initially screened and excluded were not preserved.

### Study size

No formal sample size calculation was performed because this retrospective study included all consecutive eligible patients with confirmed traumatic liver injury during the predefined study period. Therefore, the final sample size was determined by the number of eligible cases available in the hospital records.

### Clinical management definitions

Initial management was classified as NOM or initial laparotomy. NOM was defined as initial conservative management without immediate operative abdominal exploration and could include hemodynamic monitoring, serial clinical assessment, transfusion support, radiologic follow-up, and selective procedures when clinically indicated. Initial laparotomy was defined as operative abdominal exploration as the first definitive treatment strategy after presentation (1).

The initial management decision was made by the treating surgical team according to the overall clinical assessment, perceived hemodynamic stability and response to initial care, imaging findings, associated injuries, and the presence or absence of an immediate operative indication. The retrospective dataset documented the management selected but did not consistently retain admission systolic blood pressure, heart rate, shock index, lactate, base deficit, peritonitis status, or standardized resuscitation-response criteria. Hemodynamic stability therefore could not be reconstructed using a uniform numeric threshold, and the available data do not support presentation of a prospectively standardized institutional algorithm. The investigators did not influence clinical management.

Documented procedures were analyzed as a separate domain and comprised endoscopic retrograde cholangiopancreatography (ERCP) and drain placement. No hepatic angioembolization was recorded in the analytic dataset. The source dataset did not consistently distinguish image-guided from operative drain placement and did not retain the indication for, or timing of, each procedure. Accordingly, procedures were reported by type and initial management group without assigning an unrecorded indication, timing, or interventional-radiology pathway. Delayed surgery after NOM was defined as a secondary abdominal operation following an initially non-operative approach.

### Data collection and study variables

Data were extracted by one study-team member using a structured abstraction form based on predefined variables and outcome definitions. Extracted variables were based on admission records, operative notes, radiology reports, and medical charts; when sources differed, operative documentation was prioritized for intraoperative findings and radiology reports for imaging-based injury classification. Available variables included age, sex, blunt vs. penetrating mechanism, trauma cause, liver injury type and grade, specific associated abdominal and non-abdominal injury categories, Glasgow Coma Scale (GCS) score, packed red blood cell (PRBC) transfusion exposure and units, documented ERCP or drain placement, in-hospital complications, biliary leak, intensive care unit (ICU) stay, ward stay, total hospital length of stay, delayed surgery after initial NOM, and in-hospital death. Admission blood pressure, heart rate, shock index, lactate, base deficit, comorbidity burden, and standardized physiologic response to resuscitation were not captured in the retained dataset and were not imputed. Liver injury severity was classified according to the AAST liver injury scale (18), as documented in radiology reports and/or operative findings. For analysis, AAST grades I-II were categorized as lower-grade and grades III-IV as higher-grade injuries. No AAST grade V or VI injuries were identified. Associated injuries were reported both as broad abdominal/non-abdominal domains and as specific descriptive categories.

### Outcome definitions

Outcomes were assessed during the index hospitalization only. Post-discharge complications, readmissions, and late outcomes were not evaluated unless documented during the same admission episode. The primary outcome among patients initially managed non-operatively was NOM success, defined as survival without delayed operative conversion during the index hospitalization. NOM failure was defined as delayed operative conversion and/or in-hospital death after initial NOM. The broader secondary composite endpoint, termed an unfavorable NOM course, was defined as one or more of the following after initial NOM: biliary leak, any in-hospital complication, any documented ERCP or drain-placement procedure, delayed surgery, or in-hospital death. This pragmatic composite was intended to identify a clinically or resource-intensive course rather than to redefine NOM failure. Its components were reported separately, and component counts were allowed to overlap. Patients with none of these components were described as having an uncomplicated NOM course. Secondary outcomes included the individual composite components and resource-use measures, including ICU stay, ward stay, total hospital length of stay, PRBC transfusion exposure, and PRBC units. Additional exploratory analyses compared outcomes according to injury grade, mechanism, and initial treatment strategy.

### Missing data and bias

Missing data were not imputed. Available-case analysis was used for each variable, with patients excluded only from analyses requiring a missing value; denominators were reported where applicable. Because the retained dataset contained only the final eligible cohort, the number of records screened and excluded could not be reconstructed. Multiple hospital sources were used to identify cases, and a structured abstraction form with predefined definitions was used to reduce information bias. Nevertheless, data extraction by a single reviewer, lack of consistently recorded physiologic selection variables, absence of comorbidity data, and inability to reconstruct a formal selection algorithm introduce risks of abstraction bias, residual confounding, and selection bias.

### Statistical analysis

Statistical analysis was performed using IBM SPSS Statistics version 27.0 (IBM Corp., Armonk, NY, USA). Categorical variables were summarized as counts and percentages. Continuous variables were assessed by visual inspection and the Shapiro-Wilk test. Because continuous variables were non-normally distributed and several subgroup sizes were small, continuous data were reported as median and interquartile range (IQR), and between-group comparisons used the Mann–Whitney U test. Categorical comparisons used Fisher's exact test. The exact Clopper-Pearson 95% confidence interval (CI) was calculated for the observed NOM-success proportion. Comparisons between initial NOM and initial laparotomy were interpreted as descriptive and exploratory because treatment allocation was not randomized and was subject to confounding by indication. Because only six patients experienced the unfavorable-course composite, multivariable regression was not performed. Univariable analyses were treated as exploratory screening analyses rather than independent-predictor analyses. No adjustment was made for multiple comparisons; therefore, *P* values should be interpreted cautiously and alongside event counts and effect patterns. Exact two-sided *P* values were reported where appropriate, particularly for GCS because of heavy ties and small subgroup counts. All tests were two-sided, and *P* < 0.05 was considered statistically significant.

### Ethical considerations

This study was approved by the Institutional Review Board of Ibn Sina Specialized Hospital, Palestine (Reference Number: ISH-003-2025). The study was conducted in accordance with the ethical principles of the Declaration of Helsinki and all applicable institutional regulations. Because this was a retrospective review of routinely collected medical records, with no direct patient contact or intervention, the Institutional Review Board waived the requirement for individual informed consent. Medical records were accessed only by authorized members of the study team, and all extracted data were de-identified before analysis. No identifiable patient information was included in the analytic dataset or manuscript.

## Results

A total of 58 eligible patients with traumatic liver injury were included in the analytic cohort. Initial NOM was used in 48 patients (82.8%), and 10 patients (17.2%) underwent initial laparotomy. Of 27 penetrating injuries, all were gunshot wounds; no stab injuries were present. The primary NOM outcome was therefore evaluable in 48 patients. The cohort flow and outcome derivation are shown in Supplementary Figure S1 and Supplementary Table S1.

NOM success was observed in all 48 initially non-operatively managed patients (100%; exact 95% CI, 92.6%−100.0%): no patient required delayed operative conversion and no in-hospital death occurred after initial NOM. An unfavorable NOM course occurred in 6 of 48 patients (12.5%), whereas 42 patients (87.5%) had an uncomplicated NOM course. The absence of observed failure should not be interpreted as a zero underlying failure risk; the corresponding exact 95% CI for the failure proportion was 0.0%−7.4%.

Continuous variables showed non-normal distributions overall and/or within treatment groups. Accordingly, age, GCS score, ICU stay, ward stay, total length of stay, and PRBC units are summarized as median (IQR), and between-group comparisons use the Mann–Whitney U test (Supplementary Table S2). Specific associated injuries are summarized descriptively in Supplementary Table S6.

Associated injuries were common and frequently overlapped. In the full cohort, the most frequent specific abdominal injuries were renal (8/58, 13.8%), bowel (7/58, 12.1%), and splenic (6/58, 10.3%) injuries. The most frequent non-abdominal injuries were thoracic (24/58, 41.4%) and orthopedic (19/58, 32.8%) injuries. Because patients could have more than one associated injury, category percentages do not sum to 100% (Supplementary Table S6).

Among the 48 patients initially managed non-operatively, 6 (12.5%) met the composite definition of an unfavorable NOM course, whereas no patient met the definition of NOM failure. Higher-grade AAST III-IV injury was associated with an unfavorable NOM course compared with lower-grade AAST I-II injury [5/12 (41.7%) vs. 1/36 (2.8%), *P* = 0.002]. Higher-grade injury was also associated with any in-hospital complication [3/12 (25.0%) vs. 0/36 (0.0%), *P* = 0.013] and any documented procedure [3/12 (25.0%) vs. 1/36 (2.8%), *P* = 0.043], whereas biliary leak was infrequent and did not differ significantly by grade [1/12 (8.3%) vs. 0/36 (0.0%), *P* = 0.250] (Table 1; Supplementary Table S3).

**Table 1:** Outcomes among initially non-operative cases, stratified by injury grade.

Variable	Overall NOM (*n* = 48)	Lower grade (I-II) (*n* = 36)	Higher grade (III-IV) (*n* = 12)	*P* value
Clinical outcomes				
Unfavorable NOM course^a^	6/48 (12.5%)	1/36 (2.8%)	5/12 (41.7%)	0.002
Biliary leak	1/48 (2.1%)	0/36 (0.0%)	1/12 (8.3%)	0.250
Any in-hospital complication	3/48 (6.3%)	0/36 (0.0%)	3/12 (25.0%)	0.013
Any documented procedure	4/48 (8.3%)	1/36 (2.8%)	3/12 (25.0%)	0.043
Resource use, median (IQR)				
ICU stay, days	0.75 (3.0)	0.0 (1.0)	3.5 (9.8)	<0.001
Ward stay, days	3.0 (6.0)	2.0 (4.0)	8.0 (10.5)	0.001
Total length of stay, days	5.0 (10.5)	2.0 (5.9)	11.0 (22.5)	<0.001
PRBC units	0.0 (4.0)	0.0 (2.0)	4.0 (5.0)	0.020

Categorical variables are presented as *n*/*N* (%), and continuous variables as median (IQR). Fisher's exact test was used for categorical comparisons and the Mann–Whitney U test for continuous comparisons. Higher-grade refers only to AAST grades III-IV because no grade V or VI injuries were present. ^a^Composite unfavorable NOM course = any of: biliary leak, any in-hospital complication, documented ERCP or drain placement, delayed surgery, or in-hospital death. Components may overlap. No delayed surgery or in-hospital death occurred after initial NOM in either grade stratum.

Higher-grade AAST III-IV injury after initial NOM was also associated with longer ICU stay, ward stay, and total hospital stay and with greater PRBC requirements (Table 1; Supplementary Table S4). These unadjusted comparisons are exploratory and may reflect correlated differences in mechanism, associated injuries, and treatment selection.

The composite was driven by procedures and complications rather than by failure or death. Four NOM patients underwent a documented procedure (two ERCP and two drain placements), three experienced an in-hospital complication, and one developed a biliary leak. Two patients contributed to more than one component. No hepatic angioembolization was recorded. Procedure timing and indications were not available in the retained dataset; available procedure-level details are summarized in Supplementary Table S7.

Mechanism-stratified analyses showed that all 19 penetrating injuries managed with NOM were gunshot wounds. An unfavorable NOM course occurred in 6 of 19 gunshot injuries (31.6%) and in 0 of 29 blunt injuries (*P* = 0.002). Documented ERCP or drain placement was likewise confined to the gunshot subgroup [4/19 (21.1%) vs. 0/29 (0.0%), *P* = 0.020]. Exploratory screening also identified higher-grade injury, any non-abdominal injury, and any PRBC transfusion as variables associated with the composite. Because only six events occurred and these clinical features were interrelated, the analyses cannot identify independent predictors or separate the effect of mechanism from injury severity and case selection (Supplementary Tables S3, S4).

Compared with the initial-laparotomy group, patients selected for initial NOM were less likely to have a gunshot mechanism, higher-grade AAST III-IV injury, an associated abdominal injury, or PRBC transfusion and had lower PRBC utilization and shorter ICU, ward, and total hospital stays (Table 2; Supplementary Tables S3, S4). In exploratory full-cohort comparisons, initial NOM was also associated with lower rates of in-hospital complications, biliary leak, and in-hospital death (Table 3; Supplementary Table S5). These comparisons represent differences in selected case mix and outcomes, not causal estimates of treatment effectiveness.

**Table 2:** Baseline characteristics of the full cohort.

Variable	Overall (*n* = 58)	Initial NOM (*n* = 48)	Initial laparotomy (*n* = 10)	*P* value
Demographics				
Age, years	21.5 (15.0)	20.0 (14.3)	26.5 (15.8)	0.198
Male sex	50/58 (86.2%)	41/48 (85.4%)	9/10 (90.0%)	1.000
GCS, median (IQR)^b^	15.0 (0.0)	15.0 (0.0)	15.0 (0.0)	0.667
Injury profile				
Penetrating mechanism	27/58 (46.6%)	19/48 (39.6%)	8/10 (80.0%)	0.034
Higher-grade injury (III-IV)	20/58 (34.5%)	12/48 (25.0%)	8/10 (80.0%)	0.002
Associated abdominal injury	18/58 (31.0%)	10/48 (20.8%)	8/10 (80.0%)	0.001
Non-abdominal injury	35/58 (60.3%)	28/48 (58.3%)	7/10 (70.0%)	0.725
Transfusion				
Any PRBC transfusion	28/58 (48.3%)	19/48 (39.6%)	9/10 (90.0%)	0.005
PRBC units, median (IQR)	0.0 (4.0)	0.0 (4.0)	6.0 (12.0)	<0.001
Resource use				
ICU stay, days	1.5 (5.0)	0.75 (3.0)	7.5 (4.8)	<0.001
Ward stay, days	3.5 (10.3)	3.0 (6.0)	13.0 (14.0)	0.012
Total length of stay, days	6.75 (14.0)	5.0 (10.5)	16.0 (20.3)	0.001

Categorical variables: *n*/*N* (%). Continuous variables: median (IQR). Fisher exact test for categorical comparisons; Mann–Whitney U test for continuous comparisons. GCS, Glasgow Coma Scale; ICU, intensive care unit; IQR, interquartile range; NOM, non-operative management; PRBC, packed red blood cells. ^b^Exact two-sided P reported for GCS owing to heavy ties and small subgroup counts.

**Table 3:** Exploratory comparison of clinical outcomes and resource use: initial NOM vs. initial laparotomy.

Variable	Initial NOM (*n* = 48)	Initial laparotomy (*n* = 10)	*P* value
Clinical outcomes			
Any in-hospital complication	3/48 (6.3%)	5/10 (50.0%)	0.002
Biliary leak	1/48 (2.1%)	4/10 (40.0%)	0.002
In-hospital death	0/48 (0.0%)	2/10 (20.0%)	0.027
Resource use, median (IQR)			
ICU stay, days	0.75 (3.0)	7.5 (4.8)	<0.001
Ward stay, days	3.0 (6.0)	13.0 (14.0)	0.012
Total length of stay, days	5.0 (10.5)	16.0 (20.3)	0.001
PRBC units	0.0 (4.0)	6.0 (12.0)	<0.001

Table 3 is exploratory and represents a selection/outcome contrast rather than a causal effectiveness analysis. Categorical variables are presented as *n*/*N* (%) and compared using Fisher's exact test; continuous variables are presented as median (IQR) and compared using the Mann–Whitney U test. Baseline selection differences are reported in Table 2. Documented ERCP/drain procedures are described separately because their timing, indication, and clinical meaning were not equivalent across the NOM and laparotomy groups.

## Discussion

This study describes selective NOM of traumatic liver injury in a Palestinian single-center cohort. NOM was used in most patients, and no delayed operative conversion or in-hospital death was observed among the 48 patients selected for initial NOM. Nevertheless, six patients experienced an unfavorable in-hospital course, driven by procedures and complications. These events were concentrated among patients with higher-grade AAST III-IV injuries and gunshot wounds and were accompanied by greater transfusion and hospital-resource use. The findings support the feasibility of selective NOM in this setting but do not establish safety, comparative effectiveness, or a universally applicable selection algorithm.

The observed 48/48 NOM-success proportion is compatible with the high success rates reported in carefully selected cohorts (3, 9). Its precision is limited, however: the exact 95% CI was 92.6%-100.0%, and the absence of observed failures remains compatible with a true failure proportion of up to approximately 7.4%. In addition, treatment selection was made by the treating team, physiologic selection variables were not consistently retained, and follow-up ended at hospital discharge. The result should therefore be interpreted as a descriptive finding in a selected cohort rather than proof that NOM is uniformly safe or superior to operative management.

Higher-grade AAST III-IV injury was associated with an unfavorable course and greater resource use. This pattern is consistent with evidence linking increasing injury severity to hemorrhage, complications, adjunctive treatment, and NOM failure (10, 12, 13, 19). However, the present cohort contained no AAST grade V or VI injuries. Consequently, its results cannot be extrapolated to the most severe hepatic injuries, which may have different hemorrhagic risks, monitoring requirements, and thresholds for operative or endovascular intervention. The term higher-grade is therefore used throughout this article specifically for grades III-IV, rather than implying representation of the full high-grade spectrum.

All penetrating injuries in this cohort were gunshot wounds; no stab injuries were represented. Selective NOM of liver gunshot injury has been reported in carefully selected patients, but penetrating trauma presents distinct concerns regarding trajectory, associated hollow-viscus or mesenteric injury, and occult bleeding (6). Current guidance permits NOM in selected patients without an immediate operative indication when close observation and rapid escalation are possible (1). In the present study, all unfavorable NOM courses occurred after gunshot injury. This finding may reflect the combined influence of mechanism, higher injury grade, associated injuries, transfusion requirement, and selection practices; the small number of events precluded multivariable separation of these factors.

Documented procedures after initial NOM comprised two ERCP procedures and two drain placements, all in patients with gunshot injuries. Contemporary liver-trauma care may use angioembolization for arterial hemorrhage and endoscopic or drainage procedures for biliary and fluid complications (20–22). No angioembolization was recorded in this cohort. The retained dataset did not consistently document procedure indication, timing, or whether drainage was image-guided, and these details should not be inferred retrospectively. Available procedure types and clinical context are therefore reported descriptively, while the absence of timing and indication data is acknowledged as a limitation. Biliary complications remain recognized after liver trauma and may be managed using drainage and ERCP in selected cases (23–25).

Higher-grade injury was associated with longer ICU, ward, and total hospital stays and with greater transfusion requirements. Similar relationships between injury severity and resource use have been described in other liver-trauma cohorts (2, 9, 10, 13). In this study, these unadjusted associations may also reflect mechanism and associated injuries. Because detailed physiologic measures were unavailable, the analysis cannot determine how much of the observed resource use was attributable to initial shock, response to resuscitation, or liver injury grade itself.

Patients selected for initial NOM had fewer complications and shorter stays than those undergoing initial laparotomy, but the groups had markedly different baseline profiles. The laparotomy group contained higher proportions of gunshot injury, higher-grade liver injury, associated abdominal injury, and transfusion exposure. Observational comparisons of operative and non-operative groups are therefore highly vulnerable to confounding by indication (16, 26). The apparent outcome advantage of NOM should be interpreted as evidence that lower-risk patients were selected for conservative care, not as evidence of causal superiority.

The study contributes data from Palestine, a setting that is rarely represented in the liver-trauma literature. However, the retained dataset did not include a contemporaneous inventory of CT availability, ICU capacity, blood-bank resources, interventional-radiology coverage, endoscopy availability, or transfer pathways. Accordingly, institution-specific resource constraints cannot be characterized reliably from the study data. More broadly, conflict-affected and under-resourced trauma systems may face limitations in infrastructure, workforce, access, and timely referral (17); future multicenter work should characterize these system-level factors prospectively.

This study has several strengths. It provides real-world data from an under-represented Palestinian center, includes both blunt injury and firearm-related penetrating injury, reports the components of the unfavorable-course composite separately, and presents detailed injury, procedure, and resource-use information. Important limitations remain. The retrospective, single-center design and small sample limit precision and generalizability. Only six unfavorable-course events occurred, precluding robust multivariable modeling. Admission blood pressure, heart rate, shock index, lactate, base deficit, peritonitis status, standardized response to resuscitation, comorbidities, and a reproducible institutional selection algorithm were not available in the retained dataset. The timing and indication of ERCP and drain placement were also unavailable, and the dataset did not consistently distinguish image-guided from operative drainage. Counts of records screened and excluded were not retained. No AAST grade V or VI injuries and no stab injuries were represented. Multiple exploratory comparisons were performed without multiplicity adjustment. Finally, follow-up was limited to the index hospitalization, so late failure, readmission, delayed biliary complications, and functional outcomes could not be assessed.

## Conclusion

No delayed operative conversion or in-hospital death was observed among the 48 selected patients initially managed non-operatively, although the confidence interval and study limitations do not exclude a clinically relevant underlying failure risk. An unfavorable in-hospital course occurred in 12.5% and was concentrated among patients with higher-grade AAST III-IV injuries and gunshot wounds. These findings support the feasibility of selective NOM at this center while underscoring the importance of cautious patient selection, close monitoring, and timely escalation. Larger prospective multicenter studies with standardized physiologic selection criteria, detailed procedure timing and indications, representation of AAST grade V-VI injuries, and post-discharge follow-up are needed before broader safety or effectiveness conclusions can be drawn.

## Data Availability

The datasets presented in this article are not readily available because the dataset contains sensitive clinical information derived from hospital medical records and is therefore not publicly available. De-identified data may be made available from the corresponding author upon reasonable request and subject to permission from Ibn Sina Specialized Hospital. Requests to access the datasets should be directed to Ali Shakhshir, shakhshirali@gmail.com.
