# Supplementary material for: Selective non-operative management of traumatic liver injury in Palestine: a retrospective single-center cohort study
Source: Front Med (Lausanne). 2026 Sep 14;13:1878227. doi: 10.3389/fmed.2026.1878227 (PMC13616626; doi:10.3389/fmed.2026.1878227)
Supplement: Supplementary file 1 [file Data_Sheet_1.docx]

**Additional file 1. Supplementary figure and tables**

**Figure S1. Cohort composition and outcome derivation**

The retained analytic dataset contained the 58 eligible included cases; counts of records screened before eligibility assessment and excluded records were not preserved. Component counts within the unfavorable-course box overlap.

**Supplementary Table S1. Cohort composition and primary outcome derivation**

| **Item** | **Value** | **Definition / note** |
| --- | --- | --- |
| Total eligible records included | 58 | Full analytic cohort |
| Initial NOM | 48/58 (82.8%) | Initial conservative management without immediate laparotomy |
| Initial laparotomy | 10/58 (17.2%) | Operative abdominal exploration as the initial definitive strategy |
| Primary NOM outcome evaluable | 48 | NOM success/failure assessed only in the initially non-operative cohort |
| NOM success | 48/48 (100.0%); exact 95% CI, 92.6%-100.0% | Survival without delayed operative conversion during the index hospitalization |
| NOM failure | 0/48 (0.0%); exact 95% CI, 0.0%-7.4% | Delayed operative conversion and/or in-hospital death after initial NOM |
| Unfavorable NOM courseᵃ | 6/48 (12.5%) | One or more of: biliary leak, any in-hospital complication, documented ERCP or drain placement, delayed surgery, or in-hospital death |
| Uncomplicated NOM course | 42/48 (87.5%) | Absence of all unfavorable-course components |

ᵃ The unfavorable NOM course is a secondary pragmatic composite and is distinct from NOM failure. Components may overlap within the same patient. ERCP, endoscopic retrograde cholangiopancreatography; NOM, non-operative management.

**Supplementary Table S2. Distribution review for continuous variables and chosen summary statistics**

| **Variable** | **n** | **Median (IQR)** | **Shapiro–Wilk P** | **Summary / test used** |
| --- | --- | --- | --- | --- |
| Age, years | 58 | 21.5 (15.0) | .001 | Median (IQR); Mann–Whitney U |
| GCS | 46 | 15.0 (0.0) | <.001 | Median (IQR); Mann–Whitney U (ceiling effect) |
| ICU stay, days | 58 | 1.5 (5.0) | <.001 | Median (IQR); Mann–Whitney U |
| Ward stay, days | 58 | 3.5 (10.3) | <.001 | Median (IQR); Mann–Whitney U |
| Total length of stay, days | 58 | 6.75 (14.0) | <.001 | Median (IQR); Mann–Whitney U |
| PRBC units | 57 | 0.0 (4.0) | <.001 | Median (IQR); Mann–Whitney U |

Values shown for the full analytic cohort (n = 58; n = 57 for PRBC units; n = 46 for GCS). Group-specific normality checks also supported non-parametric comparisons for subsequent between-group analyses.

Abbreviations: GCS, Glasgow Coma Scale; ICU, intensive care unit; IQR, interquartile range; PRBC, packed red blood cells.

**Supplementary Table S3. Categorical comparisons and exploratory screening results**

| **Comparison** | **Outcome / variable** | **Exact P** | **Effect estimate** |
| --- | --- | --- | --- |
| ***A. Initial NOM vs initial laparotomy — baseline*** | | | |
| Initial NOM vs laparotomy | Male sex | 1.000 | 41/48 (85.4%) vs 9/10 (90.0%) |
| Initial NOM vs laparotomy | Penetrating mechanism | .034 | 19/48 (39.6%) vs 8/10 (80.0%) |
| Initial NOM vs laparotomy | Higher-grade liver injury (III–IV) | .002 | 12/48 (25.0%) vs 8/10 (80.0%) |
| Initial NOM vs laparotomy | Any associated abdominal injury | .001 | 10/48 (20.8%) vs 8/10 (80.0%) |
| Initial NOM vs laparotomy | Any non-abdominal injury | .725 | 28/48 (58.3%) vs 7/10 (70.0%) |
| Initial NOM vs laparotomy | Any PRBC transfusion | .005 | 19/48 (39.6%) vs 9/10 (90.0%) |
| ***B. Initial NOM vs initial laparotomy — outcomes*** | | | |
| Initial NOM vs laparotomy | Any in-hospital complication | .002 | 3/48 (6.3%) vs 5/10 (50.0%) |
| Initial NOM vs laparotomy | Biliary leak | .002 | 1/48 (2.1%) vs 4/10 (40.0%) |
| Initial NOM vs laparotomy | In-hospital death | .027 | 0/48 (0.0%) vs 2/10 (20.0%) |
| C. NOM cohort: lower vs higher grade | | | |
| Lower vs higher grade (NOM) | Unfavorable NOM course | .002 | 1/36 (2.8%) vs 5/12 (41.7%) |
| Lower vs higher grade (NOM) | Biliary leak | .250 | 0/36 (0.0%) vs 1/12 (8.3%) |
| Lower vs higher grade (NOM) | Any in-hospital complication | .013 | 0/36 (0.0%) vs 3/12 (25.0%) |
| Lower vs higher grade (NOM) | Any documented procedure | .043 | 1/36 (2.8%) vs 3/12 (25.0%) |
| ***D. NOM cohort: blunt vs penetrating*** | | | |
| Blunt vs penetrating (NOM) | Unfavorable NOM course | .002 | 0/29 (0.0%) vs 6/19 (31.6%) |
| Blunt vs penetrating (NOM) | Biliary leak | .396 | 0/29 (0.0%) vs 1/19 (5.3%) |
| Blunt vs penetrating (NOM) | Any in-hospital complication | .056 | 0/29 (0.0%) vs 3/19 (15.8%) |
| Blunt vs penetrating (NOM) | Any documented procedure | .020 | 0/29 (0.0%) vs 4/19 (21.1%) |
| ***E. Predictor screen within NOM cohort (outcome: unfavorable NOM course)*** | | | |
| Predictor screen (NOM) | Penetrating mechanism | .002 | 6/19 (31.6%) vs 0/29 (0.0%) |
| Predictor screen (NOM) | Higher-grade liver injury (III–IV) | .002 | 5/12 (41.7%) vs 1/36 (2.8%) |
| Predictor screen (NOM) | Any associated abdominal injury | .591 | 2/10 (20.0%) vs 4/38 (10.5%) |
| Predictor screen (NOM) | Any non-abdominal injury | .034 | 6/28 (21.4%) vs 0/20 (0.0%) |
| Predictor screen (NOM) | Any PRBC transfusion | .030 | 5/19 (26.3%) vs 1/29 (3.4%) |
| Predictor screen (NOM) | Male sex | .573 | 6/41 (14.6%) vs 0/7 (0.0%) |

All P values are two-sided Fisher exact tests. Effect estimates show n/N (%) for each group in the order listed. All penetrating injuries were firearm-related gunshot wounds; no stab injuries were represented. The procedure outcome includes documented ERCP or drain placement. A direct initial-management comparison for documented procedures was not presented because procedure timing and clinical meaning were not equivalent across groups.

Outcomes with zero events in all NOM strata (delayed operative conversion and in-hospital death) were not statistically estimable and are omitted. No delayed surgery or in-hospital death occurred after initial NOM. P values are exploratory and were not adjusted for multiple comparisons.

**Supplementary Table S4. Mann–Whitney U test results for all continuous comparisons**

| **Comparison** | **Variable** | **U statistic** | **P value** | **Median (IQR): group 1 vs group 2** |
| --- | --- | --- | --- | --- |
| ***A. Initial NOM vs initial laparotomy*** | | | | |
| NOM vs laparotomy | Age, years | 177.5 | .198 | 20.0 (14.3) vs 26.5 (15.8) |
| NOM vs laparotomy | GCS | 106.5 | .667ᵃ | 15.0 (0.0) vs 15.0 (0.0) |
| NOM vs laparotomy | ICU stay, days | 65.0 | <.001 | 0.75 (3.0) vs 7.5 (4.8) |
| NOM vs laparotomy | Ward stay, days | 119.0 | .012 | 3.0 (6.0) vs 13.0 (14.0) |
| NOM vs laparotomy | Total length of stay, days | 72.5 | .001 | 5.0 (10.5) vs 16.0 (20.3) |
| NOM vs laparotomy | PRBC units | 67.0 | <.001 | 0.0 (4.0) vs 6.0 (12.0) |
| B. NOM cohort: lower vs higher grade | | | | |
| Lower vs higher grade | ICU stay, days | 51.5 | <.001 | 0.0 (1.0) vs 3.5 (9.8) |
| Lower vs higher grade | Ward stay, days | 80.5 | .001 | 2.0 (4.0) vs 8.0 (10.5) |
| Lower vs higher grade | Total length of stay, days | 69.5 | <.001 | 2.0 (5.9) vs 11.0 (22.5) |
| Lower vs higher grade | PRBC units | 129.5 | .020 | 0.0 (2.0) vs 4.0 (5.0) |
| ***C. NOM cohort: blunt vs penetrating*** | | | | |
| Blunt vs penetrating | ICU stay, days | 110.0 | <.001 | 0.0 (1.0) vs 3.0 (4.0) |
| Blunt vs penetrating | Ward stay, days | 112.0 | <.001 | 1.0 (3.5) vs 7.0 (12.0) |
| Blunt vs penetrating | Total length of stay, days | 110.0 | <.001 | 2.0 (5.3) vs 10.0 (14.0) |
| Blunt vs penetrating | PRBC units | 103.5 | <.001 | 0.0 (0.0) vs 4.0 (6.0) |
| ***D. Predictor screen within NOM (outcome: unfavorable NOM course)*** | | | | |
| Predictor screen | Age, years | 117.5 | .796ᵃ | 20.0 (15.3) vs 20.0 (7.8) |
| Predictor screen | GCS | 82.5 | .843ᵃ | 15.0 (0.0) vs 15.0 (0.0) |

Group 1 versus Group 2 follows the order in the comparison column (e.g., NOM vs. laparotomy; lower vs. higher grade; blunt vs. penetrating; favorable vs. unfavorable NOM course for the predictor screen).

ᵃ Exact P reported owing to heavy ties or small subgroup counts.

GCS was available for 40 NOM and 6 laparotomy cases; PRBC units were available for 48 NOM and 9 laparotomy cases. All mechanism-stratified NOM comparisons used complete data for 48 cases. For the predictor screen, GCS was available for 35 favorable-course and 5 unfavorable-course NOM cases.

**Supplementary Table S5. Event-level summary of complications, biliary leak, documented procedures, and deaths**

| **Event domain** | **Total (n = 58)** | **Initial NOM (n = 48)** | **Initial laparotomy (n = 10)** | **P value (NOM vs lap)** | **P value (grade/mechanism)** |
| --- | --- | --- | --- | --- | --- |
| In-hospital complication | 8/58 (13.8%) | 3/48 (6.3%) | 5/10 (50.0%) | .002 | Grade: .013; Mechanism: .056 |
| Biliary leak | 5/58 (8.6%) | 1/48 (2.1%) | 4/10 (40.0%) | .002 | Grade: .250; Mechanism: .396 |
| Documented ERCP/drain procedure | 13/58 (22.4%) | 4/48 (8.3%) | 9/10 (90.0%) | Not comparedᵃ | Grade: .043; mechanism: .020 |
| In-hospital death | 2/58 (3.4%) | 0/48 (0.0%) | 2/10 (20.0%) | .027 | Not estimableᵇ |

Categorical variables are presented as n/N (%). Fisher exact tests are shown where clinically comparable. Procedure types are described in Table S7.

ᵃ A direct NOM-versus-laparotomy comparison was not emphasized because drain placement may have different timing and clinical meaning in the two initial-management groups and the retained dataset did not record procedure timing or indication. b No in-hospital deaths occurred after initial NOM; grade- and mechanism-stratified tests were not estimable.

Abbreviations: ERCP, endoscopic retrograde cholangiopancreatography; NOM, non-operative management.

**Supplementary Table S6. Detailed associated injury profile by initial management**

| **Injury category** | **Overall (n = 58)** | **Initial NOM (n = 48)** | **Initial laparotomy (n = 10)** |
| --- | --- | --- | --- |
| Any associated abdominal injury | 18/58 (31.0%) | 10/48 (20.8%) | 8/10 (80.0%) |
| Renal injury | 8/58 (13.8%) | 5/48 (10.4%) | 3/10 (30.0%) |
| Bowel injury | 7/58 (12.1%) | 3/48 (6.3%) | 4/10 (40.0%) |
| Splenic injury | 6/58 (10.3%) | 3/48 (6.3%) | 3/10 (30.0%) |
| Gastric injury | 2/58 (3.4%) | 0/48 (0.0%) | 2/10 (20.0%) |
| Adrenal injury | 2/58 (3.4%) | 2/48 (4.2%) | 0/10 (0.0%) |
| Mesenteric injury | 1/58 (1.7%) | 1/48 (2.1%) | 0/10 (0.0%) |
| Pancreatic injury | 1/58 (1.7%) | 0/48 (0.0%) | 1/10 (10.0%) |
| Diaphragmatic injury | 1/58 (1.7%) | 0/48 (0.0%) | 1/10 (10.0%) |
| Any associated non-abdominal injury | 35/58 (60.3%) | 28/48 (58.3%) | 7/10 (70.0%) |
| Thoracic injury | 24/58 (41.4%) | 17/48 (35.4%) | 7/10 (70.0%) |
| Orthopedic injury | 19/58 (32.8%) | 16/48 (33.3%) | 3/10 (30.0%) |
| Head or neck injury | 7/58 (12.1%) | 7/48 (14.6%) | 0/10 (0.0%) |
| Vascular injury | 2/58 (3.4%) | 1/48 (2.1%) | 1/10 (10.0%) |

Values are n/N (%). Specific injury categories are not mutually exclusive; a patient may contribute to more than one category. Categories reflect the terminology recorded in the structured study dataset. NOM, non-operative management.

**Supplementary Table S7. Available details for patients with documented ERCP or drain placement**

| **Case** | **Initial management** | **Mechanism / AAST grade** | **Documented procedure** | **Available drain detail** | **Recorded in-hospital outcomes** |
| --- | --- | --- | --- | --- | --- |
| 1 | Laparotomy | Gunshot / IV | Drain placement | Not recorded | Complication: delirium and cardiac arrest; biliary leak; death |
| 2 | Laparotomy | Gunshot / I | Drain placement | Two drains; one documented as subhepatic | Complication: delirium; biliary leak; survived |
| 3 | Laparotomy | Gunshot / III | Drain placement | Morrison pouch | No coded complication; no biliary leak; survived |
| 4 | Laparotomy | Blunt / III | Drain placement | Subhepatic, Douglas pouch, and splenic-site drains | Complication: pneumonia; no biliary leak; survived |
| 5 | Laparotomy | Gunshot / IV | Drain placement | Subdiaphragmatic and subhepatic | No coded complication; no biliary leak; survived |
| 6 | Laparotomy | Blunt / IV | ERCP and drain placement | Right and left subdiaphragmatic, subhepatic, and pelvic drains | Biliary leak; survived |
| 7 | Laparotomy | Gunshot / III | Drain placement | Splenic-bed and subdiaphragmatic drain directed toward liver injury | No coded complication; no biliary leak; survived |
| 8 | Laparotomy | Gunshot / IV | Drain placement | Subhepatic, subdiaphragmatic, and pelvic drains | No coded complication; no biliary leak; survived |
| 9 | Laparotomy | Gunshot / IV | ERCP | Not applicable | Complication: cardiac arrest; biliary leak; death |
| 10 | NOM | Gunshot / IV | ERCP | Not applicable | Biliary leak; no coded complication; survived |
| 11 | NOM | Gunshot / II | ERCP | Not applicable | No coded complication; no biliary leak; survived |
| 12 | NOM | Gunshot / IV | Drain placement | Subdiaphragmatic and left abdominal drains | Complications: sepsis, pneumonia, and ARDS; no biliary leak; survived |
| 13 | NOM | Gunshot / IV | Drain placement | Not recorded | No coded complication; no biliary leak; survived |

The retained dataset recorded procedure type and, for some drains, location. It did not consistently record the indication, time from admission, whether drain placement was image-guided or operative, or the temporal relationship between the procedure and a complication. These details are therefore not inferred. No hepatic angioembolization was recorded. AAST, American Association for the Surgery of Trauma; ARDS, acute respiratory distress syndrome; ERCP, endoscopic retrograde cholangiopancreatography; NOM, non-operative management.
